# Early diagnosis of aortic calcification through dental X-ray examination for dental pulp stones

**DOI:** 10.1038/s41598-023-45902-w

**Published:** 2023-10-30

**Authors:** Misao Ishikawa, Hiroyuki Kanzaki, Ryo Kodera, Takehiro Sekimizu, Satoshi Wada, Syunnosuke Tohyama, Tomomi Ida, Miho Shimoyama, Shugo Manase, Hiroshi Tomonari, Noriyuki Kuroda

**Affiliations:** 1 Department of Anatomy, School of Dental Medicine, Tsurumi University, 2-1-3 Tsurumi, Tsurumi-ku, Yokohama, Kanagawa Pref. 230-8501 Japan; 2 Department of Orthodontics, School of Dental Medicine, Tsurumi University, Yokohama, Japan; 3 Department of Oral and Maxillofacial Surgery, Kanazawa Medical University, Kanazawa, Japan

**Keywords:** Calcification, Vascular diseases

## Abstract

Vascular calcification, an ectopic calcification exacerbated by aging and renal dysfunction, is closely associated with cardiovascular disease. However, early detection indicators are limited. This study focused on dental pulp stones, ectopic calcifications found in oral tissues that are easily identifiable on dental radiographs. Our investigation explored the frequency and timing of these calcifications in different locations and their relationship to aortic calcification. In cadavers, we examined the association between the frequency of dental pulp stones and aortic calcification, revealing a significant association. Notably, dental pulp stones appeared prior to aortic calcification. Using a rat model of hyperphosphatemia, we confirmed that dental pulp stones formed earlier than calcification in the aortic arch. Interestingly, there were very few instances of aortic calcification without dental pulp stones. Additionally, we conducted cell culture experiments with vascular smooth muscle cells (SMCs) and dental pulp cells (DPCs) to explore the regulatory mechanism underlying high phosphate-mediated calcification. We found that DPCs produced calcification deposits more rapidly and exhibited a stronger augmentation of osteoblast differentiation markers compared with SMCs. In conclusion, the observation of dental pulp stones through X-ray examination during dental checkups could be a valuable method for early diagnosis of aortic calcification risk.

## Introduction

Extracellular matrix mineralization plays a vital role in bone and teeth. Ectopic calcification, an inappropriate biomineralization in soft tissues, often occurs due to dysregulated extracellular matrix calcification regulatory mechanisms^1^. Kidneys^2,3^, tendons^4,5^, and cardiovascular tissue^1,6,7^ are especially vulnerable to this pathological condition, resulting in impairment or restriction of biological functions.

The transformation of vascular smooth muscle cells (SMCs) into osteoblast-like cells is an important phenomenon in the development of vascular calcification^8,9^. This transformation involves the loss of smooth muscle specific gene expression in SMCs and an increase in osteoblastic differentiation markers, such as runt-related transcription factor (*Runx2*) and osteocalcin (*OCN*)^10–13^.

Vascular calcification is linked to myocardial infarction, angina pectoris, and mortality^7,14^. It can be categorized as atherosclerotic intimal calcification in the vessel intima and Menkeberg-type vascular calcification in the smooth muscle layer of the tunica media^15,16^. Menkeberg-type vascular calcification, occurring within the aortic media, directly contributes to vessel wall hardening^6,17^. Age-related changes^18^, diabetes mellitus^19^, and renal dysfunction associated with chronic kidney disease^6,18^ have been extensively studied in relation to vascular calcification. However, the condition often progresses without noticeable symptoms, leading to detection through radiographs during physical examinations or blood tests associated with other diseases in the elderly^17,20^. Unfortunately, there are limited tests or indicators for early detection of aortic calcification^20,21^.

Dental pulp stones, also known as ectopic calcification in oral tissues, particularly in the dental pulp^22–24^, can be easily identified on dental radiographs during checkups or treatment^22,24–26^. Some studies have revealed a higher prevalence of dental pulp stones in the first molar teeth of the elderly^25–27^, but the prevalence varies widely from 4 to 67%^26–28^.Factors such as trauma^29^, orthodontic treatment^30^, and dental caries^31^ have been associated with pulp stone development, and systemic diseases, including hypercalcemia^32^, kidney disease^33^, and cardiovascular disease^34^, have also been associated with their development.

The regulation of ectopic calcification involves exogenous phosphate (Pi) supplementation, which promotes osteoblast differentiation of stem and progenitor cells^35–37^. This results in phenotypic changes in SMCs, including the loss of smooth muscle–specific gene expression and upregulation of bone differentiation–related genes^38,39^. Some studies have shown that the phenotypic changes and calcification of SMCs resulting from elevated Pi levels depend on the sodium-dependent Pi cotransporters Pit-1, which can be inhibited by phosphonoformic acid^13,40^.

Dental pulp cells (DPCs), known for their immature elements^41,42^, are susceptible to transformation with disrupted systemic mineral balance control. However, the incidence and timing of dental pulp stone formation in response to such systemic changes remain unknown.

By testing our hypotheses that DPCs can undergo easier and faster transformation upon Pi stimulation compared with SMCs and that dental pulp stone formation may indicate aortic calcification, we found that dental pulp stones precede aortic calcification. Therefore, the observation of dental pulp stones through X-ray examination during dental checkups could serve as a valuable method for early diagnosis of aortic calcification risk.

## Results

### Association between dental pulp stones and aortic calcification in cadavers

**Figure 1:**
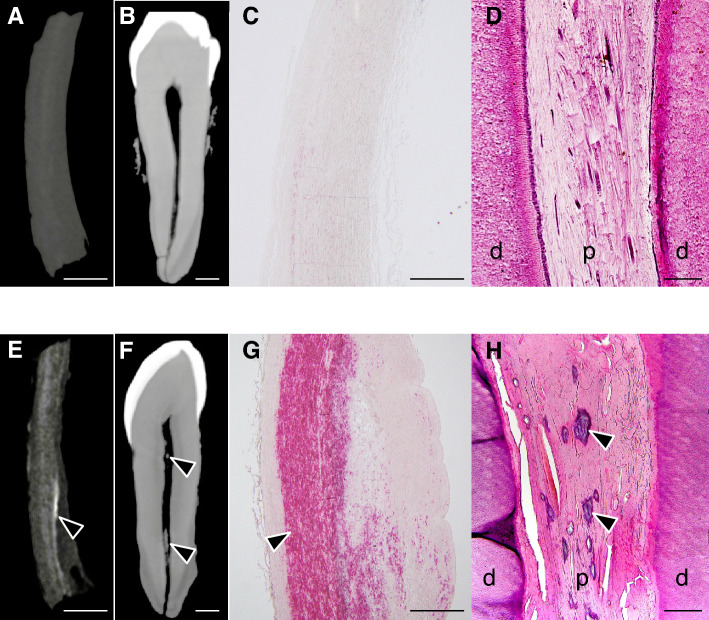
Micro-computed tomography (µCT) and histological observations of the aorta and teeth in cadavers. Representative images from cadavers (**A**–**D**) without ectopic calcification and (**E**–**H**) with ectopic calcification. µCT images of (**A**, **E**) the aortic wall and (**B**, **F**) the canine teeth in cadavers. Scale bar: 2 mm. (**C**, **G**) Alizarin Red staining of the aortic wall. Scale bar: 700 µm. (**D**, **H**) H–E staining of canine teeth. d: dentin; p: pulp. Scale bar: 200 µm. Arrowheads indicate ectopic calcification.

Histological observations confirmed that the ectopic calcifications observed on µCT images corresponded to dental pulp stones and aortic calcification of the aortic media (Fig. 1). Notably, cadavers with dental pulp stones in their canine teeth also exhibited aortic calcification of the aortic media (Fig. 1E–H). The association between these two types of ectopic calcification, was assessed using Fisher’s exact test on µCT images from 27 cadavers, with a significant result (*p* = 0.033) indicating a strong association (Table 1). Remarkably, only one out of 27 cadavers exhibited aortic calcification without dental pulp stones. These findings suggest that the formation of dental pulp stones is closely linked to aortic calcification in cadavers.

**Table 1:** Association between aortic calcification and dental pulp stone in cadavers.

Aortic calcification	Dental pulp stone		Total	*P*
	Present *n* (%)	Absent *n* (%)		
Present	13 (92.9)	1 (7.1)	14 (100.0)	0.033*
Absent	7 (53.8)	6 (46.2)	13 (100.0)	
Total	20 (74.1)	7 (25.9)	27 (100.0)	

*p < 0.05.

### Dental pulp stone formation precedes aortic calcification in DG rats

**Figure 2:**
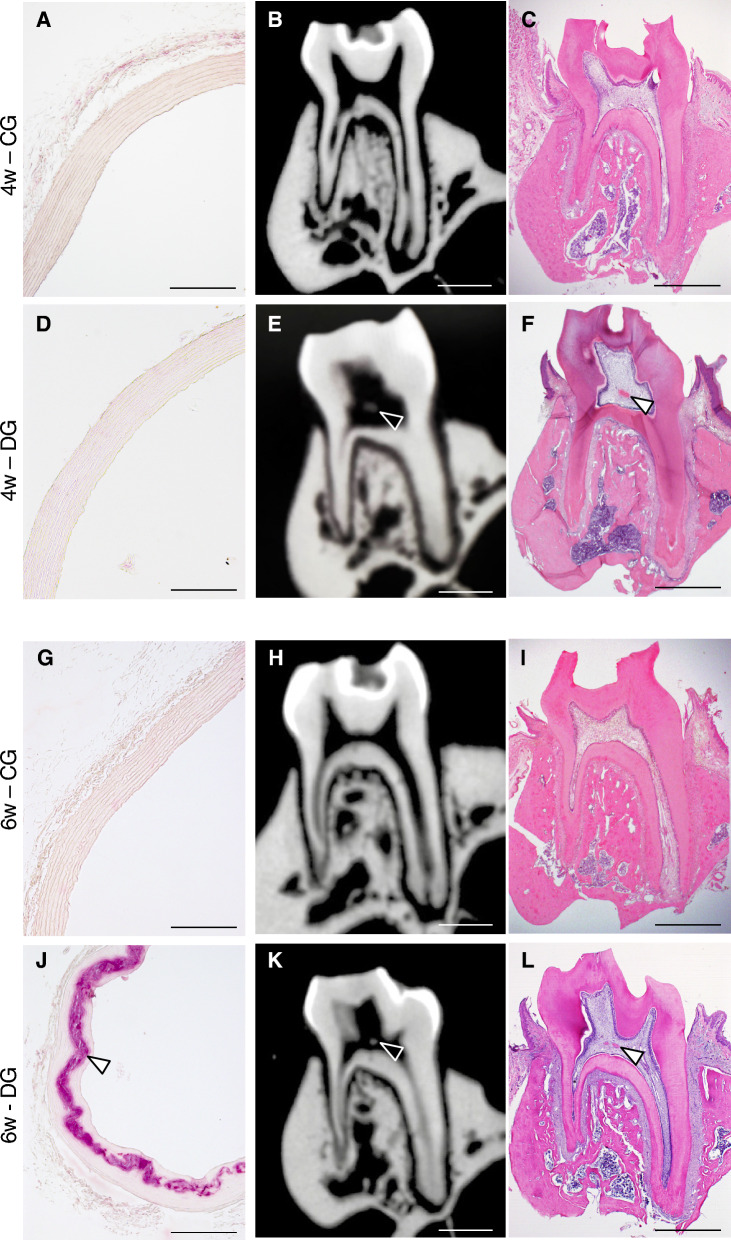
µCT images and histological observations of the aorta and first molar in rats. Representative images from rats of (**A**–**C**) the control group (CG) and (**D**–**F**) the 0.75% adenine diet group (DG) at 4 weeks. Representative images from (**G**–**I**) CG rats and (**J**–**L**) DG rats at 6 weeks. (A, D, G, J) Alizarin Red staining of cross-sections of the aortic wall. Scale bar: 200 µm. (B, E, H, K) µCT images and (C, F, I, L) H–E staining of the first molar. Scale bar: 1 mm. Arrowheads indicate ectopic calcification.

Supplemental Table S1 presents the serum parameters related to renal function and mineral metabolism. At week 6, the DG rats showed significantly higher serum Pi concentrations (21.13 mg/dL) compared with CG rats (9.32 mg/dL). µCT image and histological observations showed no calcification of both the aorta and dental pulp in the CG rats at week 4 and 6 (Fig. 2A–C,G–I). In DG rats, histological observation revealed that there was no aortic arch calcification at week 4 (Fig. 2D), whereas abundant calcification (90%) was observed at week 6 (Fig. 2J; Table 2; Supplemental Fig. S1C, D online). In contrast, dental pulp stones were already present in the first molars at week 4 and 6 in the DG rats (Fig. 2E,F,K,L). In DG rats, 90% of the molars exhibited dental pulp stones at week 4, which was a higher incidence compared with CG rats (30%) (Table 3). Notably, only one out of 10 DG rats exhibited aortic calcification without dental pulp stone formation (Table 4). These results suggest that rats on the experimental diet, which induces renal dysfunction and subsequent aortic calcification, develop dental pulp stones earlier than the onset of aortic calcification.

**Table 2:** Incidence rate of calcification in the aortic arch.

0.75% adenine diet rats	Calcification in the aortic arch		Total
	Present *n* (%)	Absent *n* (%)	
At week 4	0( 0.0)	10(100.0)	10 (100.0)
At week 6	9( 90.0)	1( 10.0)	10 (100.0)
Total	9( 45.0)	11( 55.0)	20 (100.0)

**Table 3:** Comparison of pulp stone formation between groups.

0.75% adenine diet rats	Dental pulp stone in the first morals		Total	*P*
	Present *n* (%)	Absent *n* (%)		
4w—CG	3 (30.0)	7 (70.0)	10 (100.0)	0.019*
4w—DG	9 (90.0)	1 (10.0)	10 (100.0)	
Total	12 (60.0)	8 (40.0)	20 (100.0)	

*p < 0.05.

**Table 4:** Association between aortic calcification and dental pulp stone formation in DG rats at 6 weeks.

Aortic calcification	Dental pulp stone		Total	*P*
	Present *n* (%)	Absent *n* (%)		
Present	8 ( 88.9)	1 (11.1)	9 (100.0)	0.9 (NS)
Absent	1 (100.0)	0 ( 0.0)	1 (100.0)	
Total	9 ( 90.0)	1 (10.0)	10 (100.0)	

NS: no significance, *p < 0.05.

### High-concentration Pi medium promotes calcification of dental pulp cells (DPCs)

**Figure 3:**
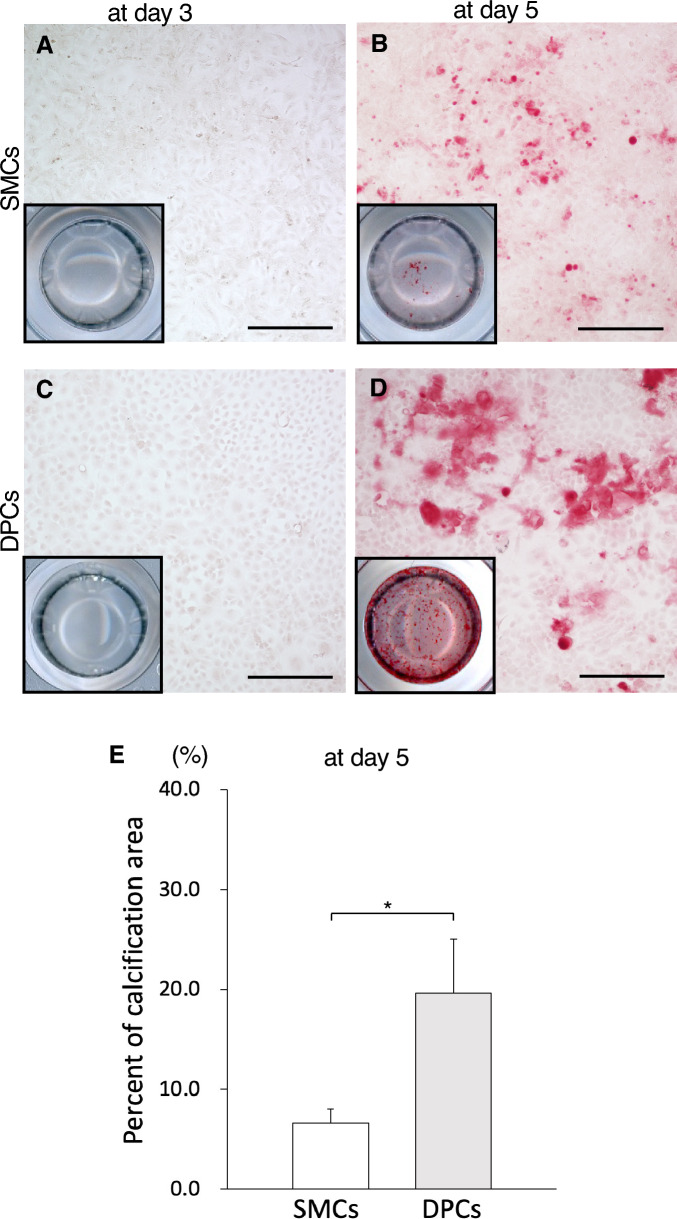
High concentrations of phosphate medium promoted calcification of DPCs. (**A**–**D**) Representative images of the cells at a higher magnification. Scale bar: 300 µm. Black box indicates a representative image of the well at a lower magnification. (**E**) Percentage of calcification area in SMCs and DPCs cultured with calcification medium supplemented with 6 mM Pi at day 5. Percentages were calculated based on the positive area of calcification per field, and results are shown as means ± standard errors of the mean (SEMs; 16–19 images in each culture condition). * *p* < 0.05 in comparisons between samples.

To investigate the mechanism underlying early calcification in dental pulp compared with the aortic media, cell culture experiments were conducted with adjusted Pi concentrations. At day 3, neither cell type showed calcium deposition (Fig. 3A,C). However, by day 5, calcium depositions were observed, particularly in DPCs (Fig. 3B,D). Measurement of the percentage of calcification area at day 5 confirmed that DPCs exhibited significantly higher calcification compared with SMCs (Fig. 3E). These findings indicate that DPCs are more sensitive to high concentrations of Pi than SMCs, leading to increased calcification.

### High-concentration Pi medium enhances expression of osteoblast differentiation markers in DPCs

**Figure 4:**
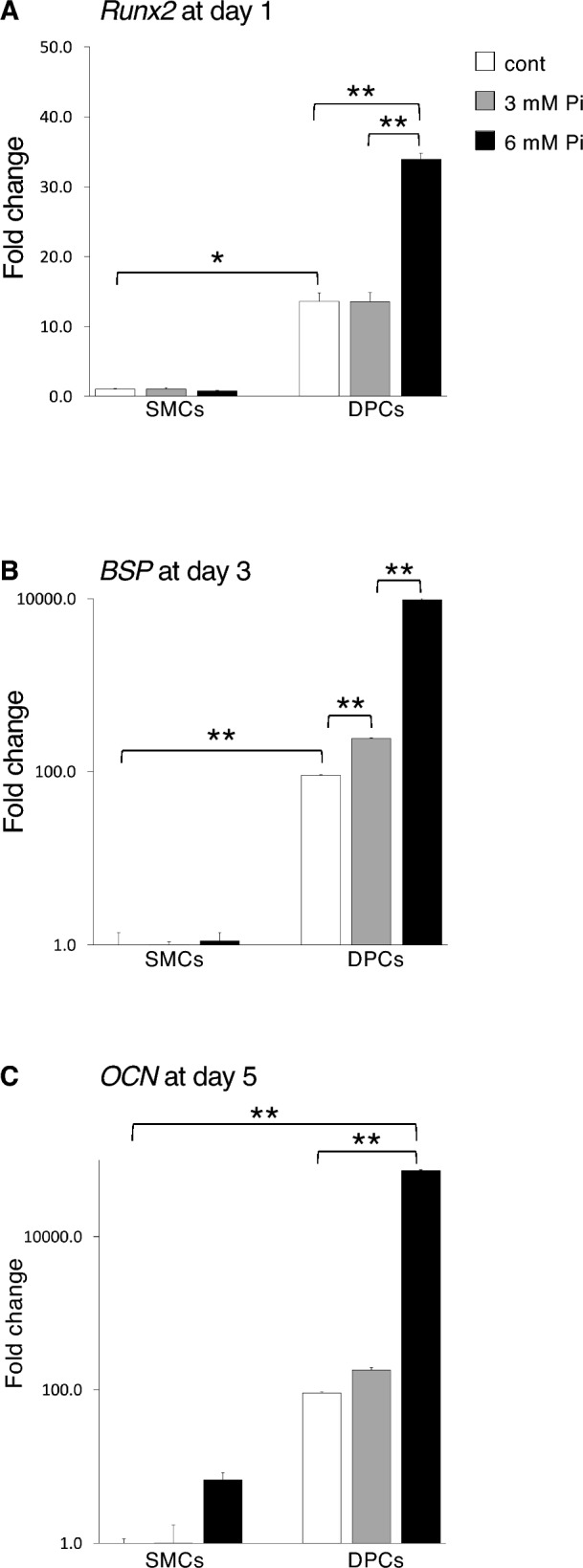
High concentrations of phosphate medium resulted in upregulation of osteoblast differentiation markers in DPCs compared with SMCs. Real-time RT-PCR analysis results for *Runx2* (**A**), *BSP* (**B**), and *OCN* (**C**) expression in SMCs and DPCs cultured in calcification medium supplemented with 3.0 and 6.0 mM Pi at day 1, 3, and 5. Biological triplicate experiments were analyzed. Fold changes in SMCs and DPCs relative to the control are shown. All values are means ± SEMs. * and ** indicate *p* < 0.05 and *p* < 0.001 in comparisons between samples, respectively. cont: cells cultured with DMEM containing 10% FBS (containing 0.9 mM Pi); 3 mM Pi: cells cultured in calcification medium supplemented with 3.0 mM Pi; 6 mM Pi: cells cultured in calcification medium supplemented with 6.0 mM Pi.

Pi medium at high concentrations influenced the phenotype of DPCs, promoting osteoblastic differentiation compared with SMCs (Fig. 4). The mRNA expression of *Runx2*, a master regulator of osteoblastic differentiation^43,44^, was significantly upregulated in DPCs cultured with calcification medium supplemented with 6 mM Pi at day 1 (Fig. 4A). In contrast, SMCs cultured with the same supplemented medium showed no change at day 1. Regarding *BSP*, an early marker of osteoblastic differentiation^45,46^, mRNA expression in DPCs cultured with calcification medium supplemented with 3 and 6 mM Pi was significantly upregulated at day 3 (Fig. 4B). *OCN*, a terminal differentiation marker for osteoblasts^47,48^, was upregulated in DPCs cultured with calcification medium supplemented with 6 mM Pi at day 5 (Fig. 4C). Collectively, these results suggest that Pi in the culture medium enhances the expression of osteoblast differentiation markers in DPCs in a time- and concentration-dependent manner.

### High-concentration Pi medium promotes nuclear translocation of Runx2 in DPCs

**Figure 5:**
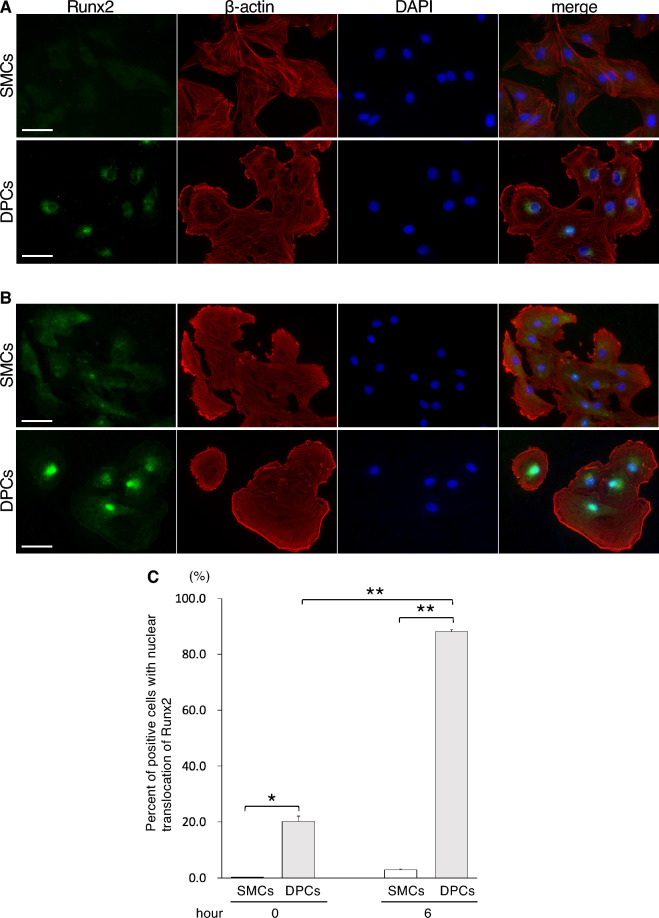
High concentrations of phosphate influenced nuclear translocation of Runx2 in DPCs. Representative immunofluorescence images from cells cultured with DMEM containing 10% FBS (**A**) and calcification medium supplemented with 6.0 mM Pi (**B**) at 6 h. Top and bottom panels show images of SMCs and DPCs, respectively. Images of Runx2, *β*-actin, DAPI, and the respective merged images are shown (from the left). Scale bar: 50 µm. (**C**) Percentage of positive cells exhibiting nuclear translocation of Runx2. The calculated values from 10 images in each culture condition are shown as means ± SEMs. * and ** indicate *p* < 0.05 and *p* < 0.001 for comparisons between groups, respectively.

To further investigate the effect of high-concentration Pi medium on osteoblastic differentiation, the nuclear translocation of Runx2 was examined through immunofluorescence staining in cells cultured with calcification medium supplemented with 6 mM Pi for 6 h. At hour 0, Runx2 expression in SMCs was not observed, whereas DPCs exhibited subtle cytoplasmic expression of Runx2 around the nucleus (Fig. 5A). After 6 h of culture in 6 mM Pi, SMCs showed faint cytoplasmic Runx2 expression (Fig. 5B). In contrast, DPCs exhibited strong expression of Runx2, predominantly located in the nuclei. Quantification of cells with nuclear translocation of Runx2 revealed that only 2.9% of SMCs showed nuclear translocation, whereas DPCs exhibited 88.1% nuclear translocation, demonstrating a significant increase compared with SMCs (Fig. 5C). These findings suggest that high-concentration Pi medium promotes the expression and nuclear translocation of Runx2 more easily in DPCs than in SMCs.

### High-concentration Pi medium increases the expression of bone sialoprotein (BSP) protein in DPCs

**Figure 6:**
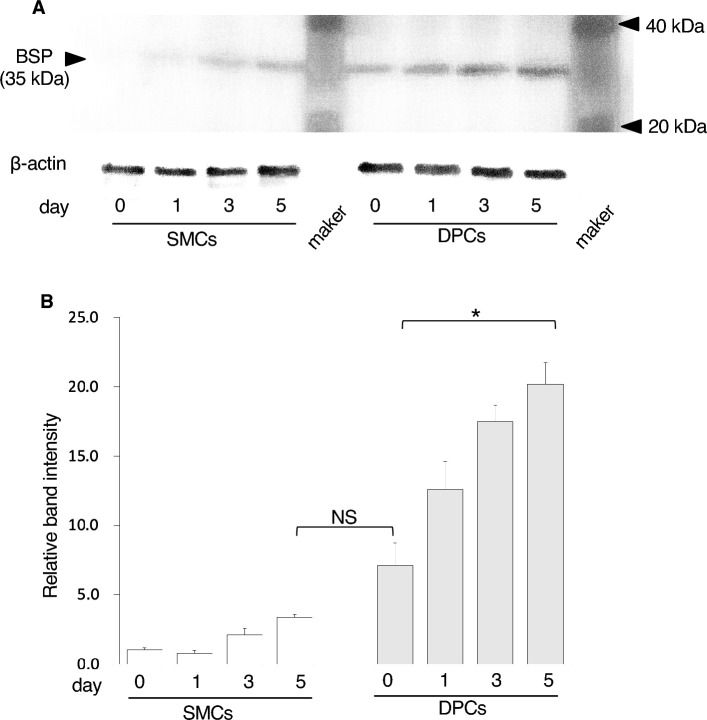
BSP production was influenced by a higher phosphate concentration, especially in DPCs. (**A**) Representative images of western blotting for BSP (top panel) and β-actin (bottom panel) from biological triplicate experiments. (**B**) Relative band intensity of western blotting for BSP normalized according to β-actin. * *p* < 0.05 for comparisons between samples. The original blot images are available online as Supplemental Figure S2.

Protein expression of BSP was examined in cells cultured with calcification medium supplemented with 6 mM Pi (Fig. 6A). Western blot analysis showed no BSP band in SMCs at day 0, whereas DPCs exhibited a band. SMCs cultured in 6 mM Pi at day 1 exhibited a faint band that became thicker over time. DPCs cultured in 6 mM Pi exhibited a time-dependent increase in band thickness, similar to SMCs, but with a distinct difference in intensity. The BSP band of SMCs at day 5 reached a similar level to that of DPCs at day 0. Densitometric analysis of the BSP band showed a time-dependent increase in intensity for both cell types (Fig. 6B). The relative band intensity of DPCs at day 0 was higher than that of SMCs at day 5. These results indicate that high-concentration Pi promotes the expression of BSP, a protein marker of osteoblast differentiation, more readily in DPCs than in SMCs.

### High-concentration Pi promotes expression of OCN protein in DPCs

**Figure 7:**
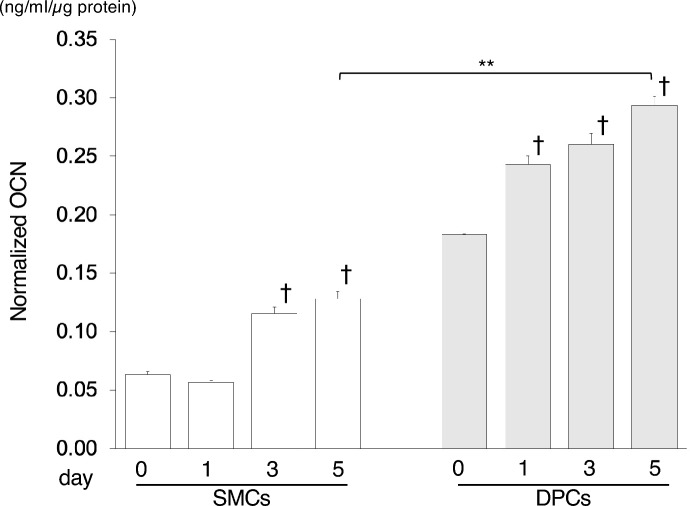
OCN production was augmented by higher phosphate concentrations in DPCs compared with SMCs. OCN concentrations in cell lysates were measured using ELISA for biological triplicate experiments. Normalized OCN levels were calculated based on total protein concentrations as the ratio of OCN/total protein (ng/ml/µg protein), and values are expressed as means ± SEMs. **p < 0.001 for comparisons between samples; † *p* < 0.001 versus SMCs or DPCs on day 0.

The production of OCN, an osteoblast differentiation marker protein^47,48^, was examined in both cell types using ELISA. High-concentration Pi significantly increased OCN concentration in a time-dependent manner (Fig. 7). Normalized OCN concentration in SMCs cultured with calcification medium supplemented with 6 mM Pi after day 1 was similar to that at day 0, although the concentration increased after day 3. Similarly, DPCs exhibited increased OCN concentration over the culture period, with a significant difference observed even at day 1 compared with day 0. Importantly, DPCs produced more OCN than SMCs, as evidenced by the distinct difference in normalized OCN concentration between the two cell types. These findings suggest that high-concentration Pi promotes OCN production, particularly in DPCs, indicating an enhanced osteoblast differentiation response.

## Discussion

In this study, we found that dental pulp stone formation precedes aortic calcification, which has implications for the early diagnosis of aortic calcification risk. Additionally, our findings indicate that DPCs and SMCs exhibit different responses to Pi concentration, with DPCs showing a greater tendency for osteoblastic cell differentiation in the presence of high Pi concentration compared with SMCs.

Previous studies have explored the association between dental pulp stones and aortic calcification^28,34,49–53^. Although some studies reported a significant association^34,49–51^, others did not find such associations^28,52,53^. However, the studies reporting associations relied on retrospective calculations of prevalence rates based on past medical history and subjective symptoms reported during interviews^28,34,51–53^. Our study overcomes these limitations by accurately assessing calcifications through µCT imaging and histological observations in cadavers, enhancing the accuracy of tissue calcification evaluation.

To the best of our knowledge, no previous studies have described the timing of formation of these ectopic calcifications. In our study, we focused on the timing of dental pulp stone and aortic calcification formation, observing a clear difference in the timing of these calcifications in rat models of hyperphosphatemia and cell culture experiments, with dental pulp stone formation preceding aortic calcification. Further experiments using more large animals such as minipig would enforce the rationale in the future^54^.

Previous studies have determined the differentiation mechanism of SMCs into osteoblast-like cells in rat models with renal damage caused by adenine administration^55–57^ and aortic calcification induced by increased serum Pi concentration^12,58,59^. However, the timing of aortic calcification and dental pulp stone formation in this rat model has not been described previously. Consistent with our findings in cadavers, our animal experiments support the earlier formation of dental pulp stones compared with aortic calcification in the aortic arch.

Previous studies have demonstrated that phenotypic transdifferentiation of SMCs promotes calcification, resulting in ectopic calcification^38,39,60^. This process involves a shift toward osteoblastic characteristics in vascular smooth muscle, characterized by decreased expression of α-SMA and SM22α^38,60^. Dental pulp calcification, however, arises from transdifferentiation of DPCs into odontoblast-like cells and subsequent calcification substance formation due to various factors, including inflammation from dental caries or abnormal occlusal pressure^23,61^.

Our results demonstrate that DPCs upregulate *Runx2* expression and increase production of osteoblastic marker proteins, such as OPN and OCN, in response to high Pi concentration, indicating their differentiation into odontoblast-like cells. Moreover, this response was significantly stronger and more rapid in DPCs compared with SMCs. These findings suggest that dental pulp stones can serve as a marker for detecting the risk of aortic calcification.

During the transdifferentiation of SMCs into osteoblast-like cells, cellular Pi uptake via the sodium-dependent Pi cotransporter Pit-1 has been reported to play a key role in phosphorylated Runx2 expression through Erk/MAPK signaling^12,62,63^. However, research on Pi transporters in oral tissues has been limited^64,65^. Our preliminary data indicate that Pit-1 is more abundantly expressed in DPCs compared with SMCs (data not shown), suggesting differential Pi uptake between the two cell types. However, further experiments are necessary to elucidate the mechanism underlying the rapid response of DPCs to Pi.

In clinical practice, the diagnosis of vascular calcification is mostly based on plain radiographs, followed by CT imaging as tool to evaluate and accurately quantify its progression^66,67^. It is also detected by indirect symptoms due to decreased compliance of the vessels such as blood pressure and pulse wave velocity^20,66,67^. These symptoms do not appear until advanced stage of the vascular calcification^17,20^. This symptom-free in early stage makes difficult to detect the vascular calcification in early stage, which leads serious limitations in curing the symptoms and restoring vascular function. Considering the limitation of early detection of the vascular calcification, there are lack of indicators that can lead to early detection of the vascular calcification in clinical practice. Here, the results of this study indicate that dental pulp calcification precedes aortic calcification, which signifying the detection of dental pulp stones can be predictive factor for the subsequent formation of aortic calcification. Therefore, the observation of dental pulp stones, which can be easily identifiable on dental radiographs, may contribute to the early diagnosis of aortic calcification.

In conclusion, our study demonstrates that dental pulp stone formation precedes aortic calcification. This finding suggests that observing dental pulp stones through dental checkups and X-ray examinations could be useful for the early diagnosis of aortic calcification risk.

## Materials and methods

### Reagents

Hematoxylin and Eosin (H–E), Alizarin Red, RIPA buffer, and dexamethasone were purchased from FUJIFILM Wako Chemicals (Tokyo, Japan). Dulbecco’s modified Eagle’s medium (DMEM) was obtained from Nacalai Tesque Inc. (Kyoto, Japan). NucleoSpin^®^ RNA was purchased from Macherey-Nagel (Düren, Germany). iScript cDNA-Supermix, SsoFast EvaGreen-Supermix, and TGX precast gel were obtained from Bio-Rad Laboratories (Hercules, CA, USA). Ascorbic acid and DAPI were purchased from Sigma-Aldrich Co. (St. Louis, MO, USA). Polyvinylidenedifluoride (PVDF) Blocking Reagent^®^ and Can Get Signal^®^ Solution-1 and -2 were obtained from Toyobo Co. Ltd. (Tokyo, Japan). ECL^TM^ Prime Western Blotting Detection Reagent was purchased from Cytiva (Tokyo, Japan).

### Cadaveric study

This study was approved by the ethics committee of Tsurumi University (approval numbers: 1322 and 1618) and was conducted in accordance with the Guidelines for the ethics committee of Tsurumi University Regulations. The cadavers used were provided for anatomical practice to the Department of Anatomy, Tsurumi University. Written informed consent was obtained from the living cadaver donors and their families.

The cadavers with a past history of diabetes, hypertension, heart disease, or kidney problems from the application form written by the living cadaver donors were excluded. The canine teeth were chosen, because Asian people have the highest rate of canine tooth survival^68^. The untreated canine teeth in different locations were extracted using dental forceps. There were no cadavers with good occlusal relationship because the teeth were partially remaining. The male cadavers were chosen, because there are reports that hormonal changes in women affect aortic calcification formation^69^. In total, 27 untreated canine teeth and aortae were obtained from 27 formalin-fixed Japanese cadavers (males, aged 67–94 years) following a student dissection course of anatomy at Tsurumi University. The aortic samples were isolated from an area measuring 10 × 10 mm after dividing the subclavian artery in the aortic arch (Supplemental Fig. S3A online). Both the canine teeth and aortae from the cadavers were stored in 10% formalin.

### Micro-computed tomography (micro-CT) analysis of tooth and aortic samples

X-ray micro-computed tomography (µCT) analysis was conducted to detect the formation of aortic calcification and dental pulp stones. The tooth and aortic samples were scanned using a µCT system (inspeXio SMX-225 CT; Shimadzu, Kyoto, Japan) with a tube voltage of 125 kV and tube current of 70 μA. Subsequently, 3D-Bon software (Ratoc System Engineering, Tokyo, Japan) was used for the detection of dental pulp stones and aortic calcifications through image analysis.

### Histological examinations of tooth and aortic samples

Following µCT analysis, decalcified teeth and aortae were subjected to histological examination. The samples were dehydrated in a graded series of ethanol, embedded in paraffin, and cut into serial cross Sects. (8 μm in thickness). H–E and Alizarin Red staining were performed on the sections.

### Experimental animals

This study was approved by the Institutional Animal Care and Use Committee of Tsurumi University (approval numbers: 20A022 and 23A027), and performed in accordance with the Regulation Concerning for Animal Experiments at Tsurumi University. Male Wistar rats (8 weeks old; *n* = 40) obtained from CLEA Japan, Inc. (Tokyo, Japan) were housed under a 12 h light/dark cycle with ad libitum access to food and deionized water throughout the experiment. After a 2-week acclimation period, the rats were divided into four groups (*n* = 10 per group): control group (CG) and adenine diet group (DG) for 4 and 6 weeks. The CG rats were fed a normal powder diet (CE-2), whereas the DG rats were fed a CE-2 diet supplemented with 0.75% adenine^56,57^.

On the final day of the experiment, blood samples were collected via direct heart puncture from anesthetized rats for routine biochemical assays, including serum urea nitrogen, creatinine, Pi, and calcium levels (SRL Inc., Tokyo, Japan). Subsequently, a solution of 1.0% heparin sodium in 0.1 M Pi buffer was perfused through the left ventricle for approximately 60 s, followed by perfusion with 4% paraformaldehyde in 0.1 M Pi buffer (pH 7.4) for 20 min at room temperature. After perfusion, the samples (maxilla with molars and aorta) were dissected and immersed in 4% paraformaldehyde overnight. The region of interest in the aorta was defined as the area after the divergence into the subclavian artery in the aortic arch (Supplemental Fig. S3B online).

### µCT analysis of animal samples

X‐ray µCT analysis for the animal experiments was performed similarly to the cadaveric study with a minor modification: aortic calcification was analyzed using a tube voltage of 50 kV and tube current of 30 μA.

### Histological examinations of animal samples

The samples of maxilla which had been decalcified in a 10% EDTA solution (pH 7.4) for 3 weeks and the aorta were dehydrated in a graded ethanol series and processed for paraffin embedding. Serial cross sections of the aorta (5 μm in thickness) were stained with Alizarin Red, whereas serial frontal sections of the maxilla (6 μm in thickness) were stained with H–E.

### Cell culture

Rat aortic SMCs derived from the thoracic aorta were obtained from CELL Applications Inc. (CA, USA). Rat DPCs were isolated from the maxillary incisors of 10-week-old male Wistar rats (*n* = 6) using an aseptic technique. Both SMCs and DPCs were cultured in DMEM supplemented with 10% fetal bovine serum (FBS), 100 U/mL penicillin, and 100 mg/mL streptomycin. The cells were incubated at 37 °C in a 5% CO_2_ incubator, with medium changes every 3 days. The DMEM used in the culture contained 0.9 mmol/L PO_4_^3−^.

### Calcification induction assay

SMCs and DPCs were seeded in 96-well plates and subcultured to confluence, reaching a total protein content of 277–289 μg/well. Calcification was induced by switching the cells to calcification medium, which consisted of DMEM supplemented with 10% FBS, 0.01 μM dexamethasone, and 50 μg/mL ascorbic acid. The cells were further cultured for 5 days in either the standard calcification medium or the calcification medium with Pi concentrations of 3.0 and 6.0 mM (adjusted using a mixture of NaH_2_PO_4_ and Na_2_HPO_4_). The Pi concentrations in the culture medium were based on the serum Pi concentrations observed in the animal experiment (see Supplemental Table S1 online).

### Calcification assay

After the culture period, the cells were fixed with methanol and stained with Alizarin Red for 15 min. The wells were then washed with milli-Q water, and images were captured using a BZ-9000 microscope (Keyence, Osaka, Japan). The percentage of calcification area per field was calculated from 16–19 photographs for each culture condition using Image-J software (National Institutes of Health, Bethesda, MD, USA).

### RNA extraction and reverse transcription

RNA was extracted from the cultured cells using a NucleoSpin® RNA Kit with on-column genomic DNA digestion, following the manufacturer’s instructions. The concentration of the isolated RNA was measured, and 200 ng of RNA from each sample was reverse transcribed using iScript cDNA-Supermix. The resulting cDNA was diluted (2 ×) with Tris–EDTA buffer.

### Real-time reverse transcription polymerase chain reaction analysis

Real-time reverse transcription polymerase chain reaction (RT-PCR) was performed using SsoFast EvaGreen-Supermix. The primer sequences for rat β-actin, *Runx2*, *bone sialoprotein* (*BSP*), and *osteocalcin* (*OCN*) can be found in Supplemental Table S2 online. The fold changes of the genes of interest were calculated using the ∆∆Ct method, with *β-actin* serving as the reference gene.

### Immunofluorescent staining

SMCs and DPCs were seeded onto coverslips (Matsunami Glass Ind., Ltd., Osaka, Japan) in 6-well plates and treated with the calcification medium containing a Pi concentration of 6.0 mM for 6 h. Cells in the CG were cultured in DMEM supplemented with 10% FBS. After cultivation, the cells were fixed with ice-cold methanol for 20 min and washed with phosphate-buffered saline with Tween (PBS-T) at least three times. Subsequently, the cells were blocked with 10% bovine serum albumin in PBS for 1 h at room temperature. Primary antibodies, including rabbit IgG anti-Runx2 (1:300; Affinity Biosciences, OH, USA) and mouse IgG anti-β-actin (1:700; Cell Signaling Technology, MA, USA), were incubated with the cells overnight at 4℃. Following primary antibody incubation, Alexa Fluor 488—and 568–labeled secondary antibodies (1:1000; Abcam, Cambridge, UK) were added and incubated for 1 h at room temperature. The cells were then stained with DAPI (1 µg/ml) for 10 min at room temperature to visualize the nuclei. Finally, coverslips were mounted onto slides and imaged using a BZ-9000 fluorescence microscope (Keyence) with consistent exposure conditions.

### Western blot analysis

The cultured cells were lysed in RIPA buffer following the manufacturer’s instructions. The total protein content in the samples was quantified using a bicinchoninic acid protein assay (Thermo Fisher Scientific, MA, USA). Protein concentrations were adjusted using RIPA buffer. The samples were then mixed with sample buffer, heat denatured, electrophoresed on a TGX precast gel, and transferred to a PVDF membrane using a Trans-Blot® Turbo™ system (Bio-Rad Laboratories). After washing, the membrane was blocked with PVDF Blocking Reagent®, incubated with anti-BSP antibody (Affinity Biosciences) in Can Get Signal® Solution-1, and then washed with PBS-T. Subsequently, the membrane was incubated with HRP-conjugated secondary antibody (R&D Systems, MN, USA) in Can Get Signal® Solution-2 and washed with PBS-T. Chemiluminescence was generated using ECL™ Prime Western Blotting Detection Reagent and detected with a LumiCube (Liponics, Tokyo, Japan). The monochrome mode images were processed from the RGB images taken with LumiCube (see Supplemental Fig. S2 online). Densitometric analysis for BSP was performed using Image-J software.

### Enzyme-linked immunosorbent assay

The concentration of osteocalcin in the cell lysates was measured using a commercially available enzyme-linked immunosorbent assay (ELISA) kit according to the manufacturer’s instructions (Rat Osteocalcin ELISA Kit; Novus Biologicals LLC, CO, USA). The optical density of each well was determined at 370 nm using a microplate reader (BioTek Japan, Tokyo, Japan). The normalized osteocalcin per total protein was calculated as ng/ml/µg protein.

### Statistical analysis

Fisher’s exact test was used to calculate the probability (*p-value*) to assess the relationship between aortic calcification and dental pulp stones. Multiple comparisons [a priori comparisons^70^] were performed by using the Tukey test. Mean values between two groups were compared using the t-test. Statistical significance was defined as *p* < 0.05 and *p* < 0.001.

### Supplementary Information

Supplementary Information 1.

Supplementary Information 2.

Supplementary Information 3.

Supplementary Information 4.

Supplementary Information 5.

Supplementary Information 6.

Supplementary Information 7.

Supplementary Information 8.

Supplementary Information 9.

## Data Availability

The data that support the findings of this study are available from the corresponding author upon reasonable request.

## References

[1] Giachelli CM (1999). Ectopic calcification: Gathering hard facts about soft tissue mineralization. Am. J. Pathol..

[2] Alexander RT (2012). Kidney stones and kidney function loss: a cohort study. BMJ.

[3] Thongprayoon C, Krambeck AE, Rule AD (2020). Determining the true burden of kidney stone disease. Nat. Rev. Nephrol..

[4] Riley GP, Harrall RL, Constant CR, Cawston TE, Hazleman BL (1996). Prevalence and possible pathological significance of calcium phosphate salt accumulation in tendon matrix degeneration. Ann. Rheum. Dis..

[5] Catapano M, Robinson DM, Schowalter S, McInnis KC (2022). Clinical evaluation and management of calcific tendinopathy: an evidence-based review. J. Osteopath. Med..

[6] McCullough PA, Agrawal V, Danielewicz E, Abela GS (2008). Accelerated atherosclerotic calcification and Monckeberg's sclerosis: a continuum of advanced vascular pathology in chronic kidney disease. Clin. J. Am. Soc. Nephrol..

[7] Rennenberg RJ (2009). Vascular calcifications as a marker of increased cardiovascular risk: a meta-analysis. Vasc Health Risk Manag.

[8] Shioi A (1995). Beta-glycerophosphate accelerates calcification in cultured bovine vascular smooth muscle cells. Arterioscler. Thromb. Vasc. Biol..

[9] Mori K, Shioi A, Jono S, Nishizawa Y, Morii H (1999). Dexamethasone enhances In vitro vascular calcification by promoting osteoblastic differentiation of vascular smooth muscle cells. Arterioscler. Thromb. Vasc. Biol..

[10] Li, W. et al. SIRT6 protects vascular smooth muscle cells from osteogenic transdifferentiation via Runx2 in chronic kidney disease. J Clin Invest **132** (2022) 10.1172/JCI150051 PMC8718147 34793336

[11] Takemura A (2011). Sirtuin 1 retards hyperphosphatemia-induced calcification of vascular smooth muscle cells. Arterioscler. Thromb. Vasc. Biol..

[12] Mune S (2009). Mechanism of phosphate-induced calcification in rat aortic tissue culture: possible involvement of Pit-1 and apoptosis. Clin. Exp. Nephrol..

[13] Li X, Yang HY, Giachelli CM (2008). BMP-2 promotes phosphate uptake, phenotypic modulation, and calcification of human vascular smooth muscle cells. Atherosclerosis.

[14] Volgman AS (2018). Atherosclerotic cardiovascular disease in South Asians in the United States: epidemiology, risk factors, and treatments: A scientific statement from the American heart association. Circulation.

[15] Chen NX, Moe SM (2012). Vascular calcification: pathophysiology and risk factors. Curr. Hypertens. Rep..

[16] Shanahan CM, Cary NR, Metcalfe JC, Weissberg PL (1994). High expression of genes for calcification-regulating proteins in human atherosclerotic plaques. J. Clin. Invest..

[17] Dart AM (1991). Aortic distensibility in patients with isolated hypercholesterolaemia, coronary artery disease, or cardiac transplant. Lancet.

[18] Shanahan CM (2013). Mechanisms of vascular calcification in CKD-evidence for premature ageing?. Nat. Rev. Nephrol..

[19] Yahagi K (2017). Pathology of human coronary and carotid artery atherosclerosis and vascular calcification in diabetes mellitus. Arterioscler. Thromb. Vasc. Biol..

[20] Tousoulis D (2013). Serum osteoprotegerin and osteopontin levels are associated with arterial stiffness and the presence and severity of coronary artery disease. Int. J. Cardiol..

[21] Kadoglou NP (2008). The relationship between serum levels of vascular calcification inhibitors and carotid plaque vulnerability. J. Vasc. Surg..

[22] Huang LG, Chen G (2016). A histological and radiographic study of pulpal calcification in periodontally involved teeth in a Taiwanese population. J. Dental Sci..

[23] Ricucci D, Loghin S, Niu LN, Tay FR (2018). Changes in the radicular pulp-dentine complex in healthy intact teeth and in response to deep caries or restorations: A histological and histobacteriological study. J. Dent..

[24] Goga R, Chandler NP, Oginni AO (2008). Pulp stones: a review. Int. Endod. J..

[25] Kalaji MN, Habib AA, Alwessabi M (2017). Radiographic assessment of the prevalence of pulp stones in a Yemeni population sample. Eur. Endod. J..

[26] Gulsahi A, Cebeci AI, Ozden S (2009). A radiographic assessment of the prevalence of pulp stones in a group of Turkish dental patients. Int. Endod. J..

[27] Ranjitkar S, Taylor JA, Townsend GC (2002). A radiographic assessment of the prevalence of pulp stones in Australians. Aust. Dent. J..

[28] Sener S, Cobankara FK, Akgunlu F (2009). Calcifications of the pulp chamber: Prevalence and implicated factors. Clin. Oral Investig..

[29] McCabe PS, Dummer PM (2012). Pulp canal obliteration: An endodontic diagnosis and treatment challenge. Int. Endod. J..

[30] Lazzaretti DN (2014). Histologic evaluation of human pulp tissue after orthodontic intrusion. J. Endod..

[31] Bjorndal L, Darvann T (1999). A light microscopic study of odontoblastic and non-odontoblastic cells involved in tertiary dentinogenesis in well-defined cavitated carious lesions. Caries Res..

[32] Nikiforuk G, Fraser D, Poyton HG, McKendry JB (1981). Calcific bridging of dental pulp caused by iatrogenic hypercalcemia. Report of a case. Oral Surg. Oral Med. Oral Pathol..

[33] Nasstrom K, Forsberg B, Petersson A, Westesson PL (1985). Narrowing of the dental pulp chamber in patients with renal diseases. Oral Surg. Oral Med. Oral Pathol..

[34] Edds AC (2005). Pilot study of correlation of pulp stones with cardiovascular disease. J. Endod..

[35] Roberts SJ (2014). Humanized culture of periosteal progenitors in allogeneic serum enhances osteogenic differentiation and in vivo bone formation. Stem. Cells Transl. Med..

[36] Gronowicz G, Woodiel FN, McCarthy MB, Raisz LG (1989). In vitro mineralization of fetal rat parietal bones in defined serum-free medium: effect of beta-glycerol phosphate. J. Bone Miner. Res..

[37] Bellows CG, Heersche JN, Aubin JE (1992). Inorganic phosphate added exogenously or released from beta-glycerophosphate initiates mineralization of osteoid nodules in vitro. Bone Miner..

[38] Steitz SA (2001). Smooth muscle cell phenotypic transition associated with calcification: upregulation of Cbfa1 and downregulation of smooth muscle lineage markers. Circ. Res..

[39] Cai T (2016). WNT/beta-catenin signaling promotes VSMCs to osteogenic transdifferentiation and calcification through directly modulating Runx2 gene expression. Exp. Cell Res..

[40] Jono S (2000). Phosphate regulation of vascular smooth muscle cell calcification. Circ. Res..

[41] Magloire H, Joffre A, Bleicher F (1996). An in vitro model of human dental pulp repair. J. Dent. Res..

[42] Melin M (2000). Effects of TGFbeta1 on dental pulp cells in cultured human tooth slices. J. Dent. Res..

[43] Otto F (1997). Cbfa1, a candidate gene for cleidocranial dysplasia syndrome, is essential for osteoblast differentiation and bone development. Cell.

[44] Komori T (1997). Targeted disruption of Cbfa1 results in a complete lack of bone formation owing to maturational arrest of osteoblasts. Cell.

[45] Bianco P, Fisher LW, Young MF, Termine JD, Robey PG (1991). Expression of bone sialoprotein (BSP) in developing human tissues. Calcif. Tissue Int..

[46] Chen J, Shapiro HS, Sodek J (1992). Development expression of bone sialoprotein mRNA in rat mineralized connective tissues. J. Bone Miner. Res..

[47] Hauschka PV, Lian JB, Cole DE, Gundberg CM (1989). Osteocalcin and matrix Gla protein: vitamin K-dependent proteins in bone. Physiol. Rev..

[48] Javed A (1999). Multiple Cbfa/AML sites in the rat osteocalcin promoter are required for basal and vitamin D-responsive transcription and contribute to chromatin organization. Mol. Cell. Biol..

[49] Ezoddini-Ardakani F (2011). Association of pulp stones with coronary artery stenosis. Commun. Dent. Health.

[50] Khojastepour L, Bronoosh P, Khosropanah S, Rahimi E (2013). Can dental pulp calcification predict the risk of ischemic cardiovascular disease?. J. Dent Tehran.

[51] Mathew ST, Al-Mutlaq MA, Al-Eidan RF, Al-Khuraisi DM, Adam H (2019). Prevalence of pulp stones and its relation with cardiovascular diseases and diabetes mellitus using digital radiographs: a retrospective study. Ann. Dent. Spec..

[52] Horsley SH (2009). Prevalence of carotid and pulp calcifications: A correlation using digital panoramic radiographs. Int. J. Comput. Assist. Radiol. Surg..

[53] Alsweed A, Farah R, Ps S, Farah R (2019). The prevalence and correlation of carotid artery calcifications and dental pulp stones in a Saudi Arabian population. Diseases.

[54] Broekhuizen LN (2010). Effect of sulodexide on endothelial glycocalyx and vascular permeability in patients with type 2 diabetes mellitus. Diabetologia.

[55] Diwan V, Brown L, Gobe GC (2018). Adenine-induced chronic kidney disease in rats. Nephrol. Carlton.

[56] Yokozawa T, Oura H, Okada T (1982). Metabolic effects of dietary purine in rats. J. Nutr. Sci. Vitaminol. Tokyo.

[57] Sueyoshi M (2019). Effects of lactulose on renal function and gut microbiota in adenine-induced chronic kidney disease rats. Clin. Exp. Nephrol..

[58] Suzuki A (2006). Enhanced expression of the inorganic phosphate transporter Pit-1 is involved in BMP-2-induced matrix mineralization in osteoblast-like cells. J. Bone Miner. Res..

[59] Giachelli CM, Speer MY, Li X, Rajachar RM, Yang H (2005). Regulation of vascular calcification: roles of phosphate and osteopontin. Circ Res.

[60] Speer MY (2009). Smooth muscle cells give rise to osteochondrogenic precursors and chondrocytes in calcifying arteries. Circ. Res..

[61] Cao Y (2015). Pulp-dentin regeneration: Current state and future prospects. J. Dent. Res..

[62] Lau WL, Festing MH, Giachelli CM (2010). Phosphate and vascular calcification: Emerging role of the sodium-dependent phosphate co-transporter PiT-1. Thromb. Haemost..

[63] Bon N (2018). Phosphate (P(i))-regulated heterodimerization of the high-affinity sodium-dependent P(i) transporters PiT1/Slc20a1 and PiT2/Slc20a2 underlies extracellular P(i) sensing independently of P(i) uptake. J. Biol. Chem..

[64] Tada H, Nemoto E, Foster BL, Somerman MJ, Shimauchi H (2011). Phosphate increases bone morphogenetic protein-2 expression through cAMP-dependent protein kinase and ERK1/2 pathways in human dental pulp cells. Bone.

[65] Ishikawa M, Matsuzawa A, Itohiya K, Nakamura Y (2018). Phosphate through the sodium-dependent phosphate cotransporters, Pit-1 and Pit-2 is the key factor of periodontal ligament calcification. J. Hard Tissue Biol..

[66] Raggi P, Bellasi A (2007). Clinical assessment of vascular calcification. Adv. Chronic. Kidney Dis..

[67] Tolle M, Reshetnik A, Schuchardt M, Hohne M, van der Giet M (2015). Arteriosclerosis and vascular calcification: causes, clinical assessment and therapy. Eur. J. Clin. Invest..

[68] Song IS, Han K, Choi YJ, Ryu JJ, Park JB (2016). Influence of oral health behavior and sociodemographic factors on remaining teeth in Korean adults 2010–2012 Korea national health and nutrition examination survey. Med. Baltimore.

[69] Subramanya V (2019). Association of endogenous sex hormone levels with coronary artery calcium progression among post-menopausal women in the multi-ethnic study of atherosclerosis (MESA). J. Cardiovasc. Comput. Tomogr..

[70] Ruxton GD, Beauchamp G (2008). Time for some a priori thinking about post hoc testing. Behav. Ecol..

