# Supplementary figures and images for: Early diagnosis of aortic calcification through dental X-ray examination for dental pulp stones
Source: Sci Rep. 2023 Oct 30;13:18576. doi: 10.1038/s41598-023-45902-w (PMC10616172; doi:10.1038/s41598-023-45902-w)

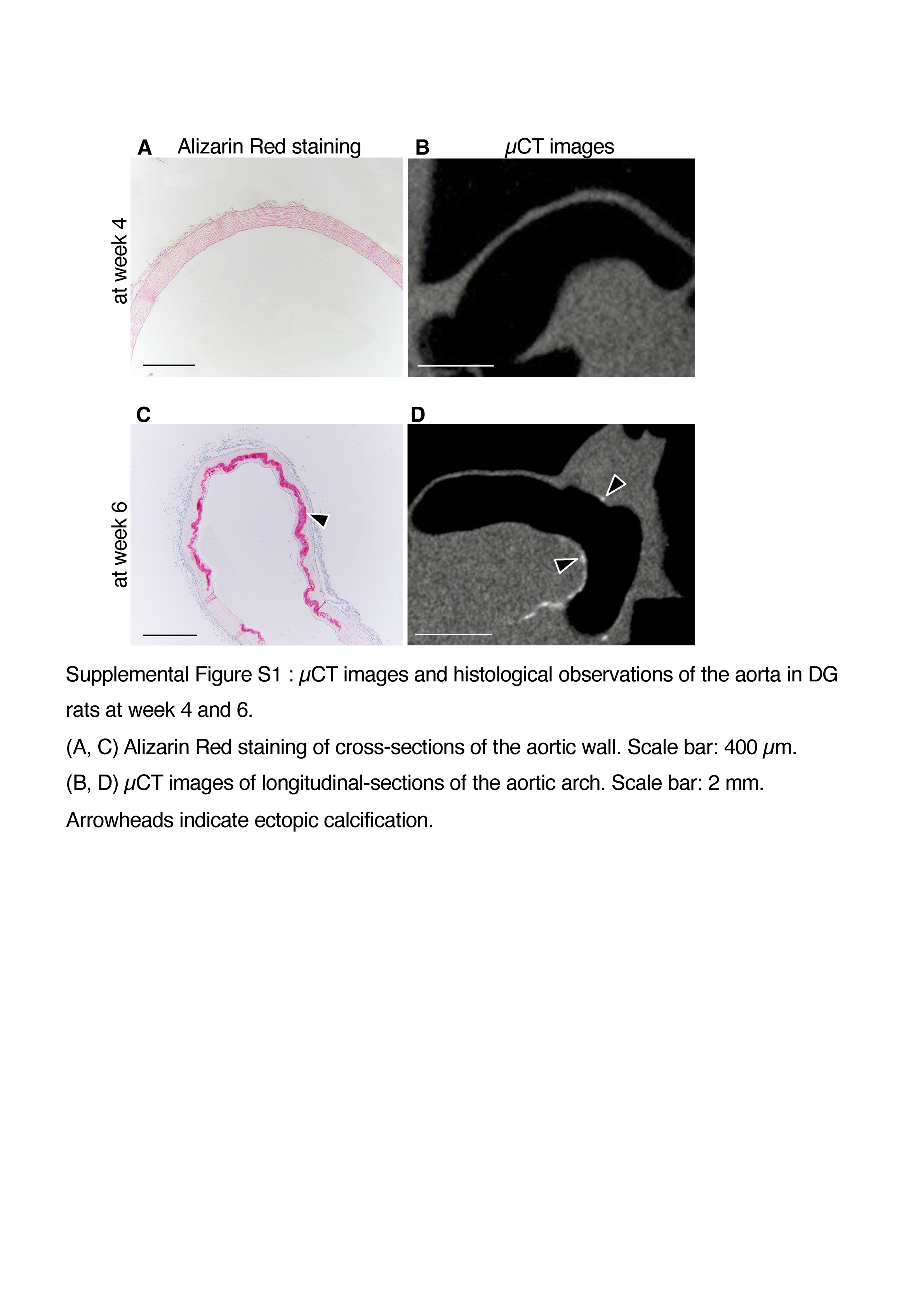

Supplement: Supplementary file 1 — Supplementary Information 1. [file 41598_2023_45902_MOESM1_ESM.tif]

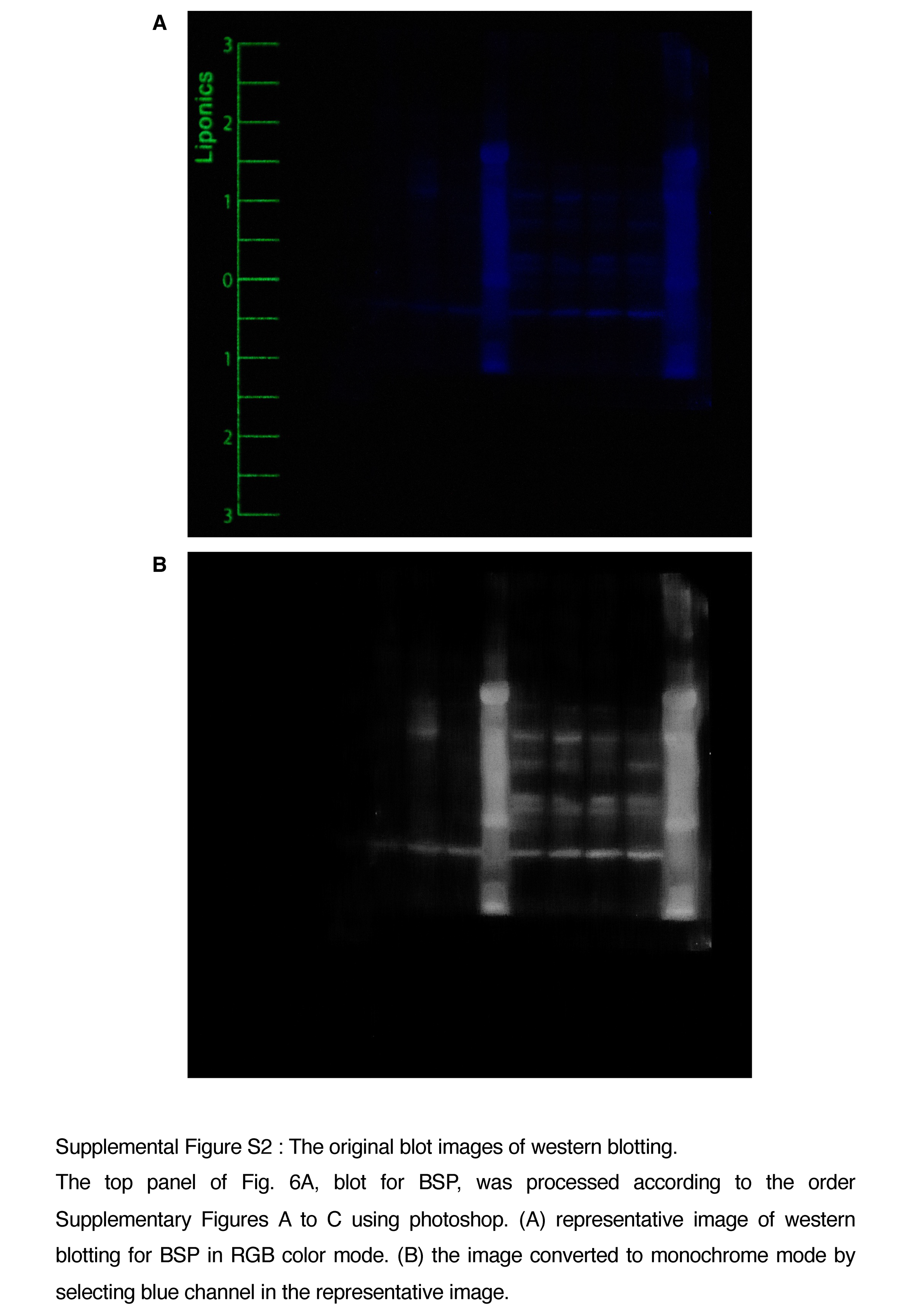

Supplement: Supplementary file 2 — Supplementary Information 2. [file 41598_2023_45902_MOESM2_ESM.tif]

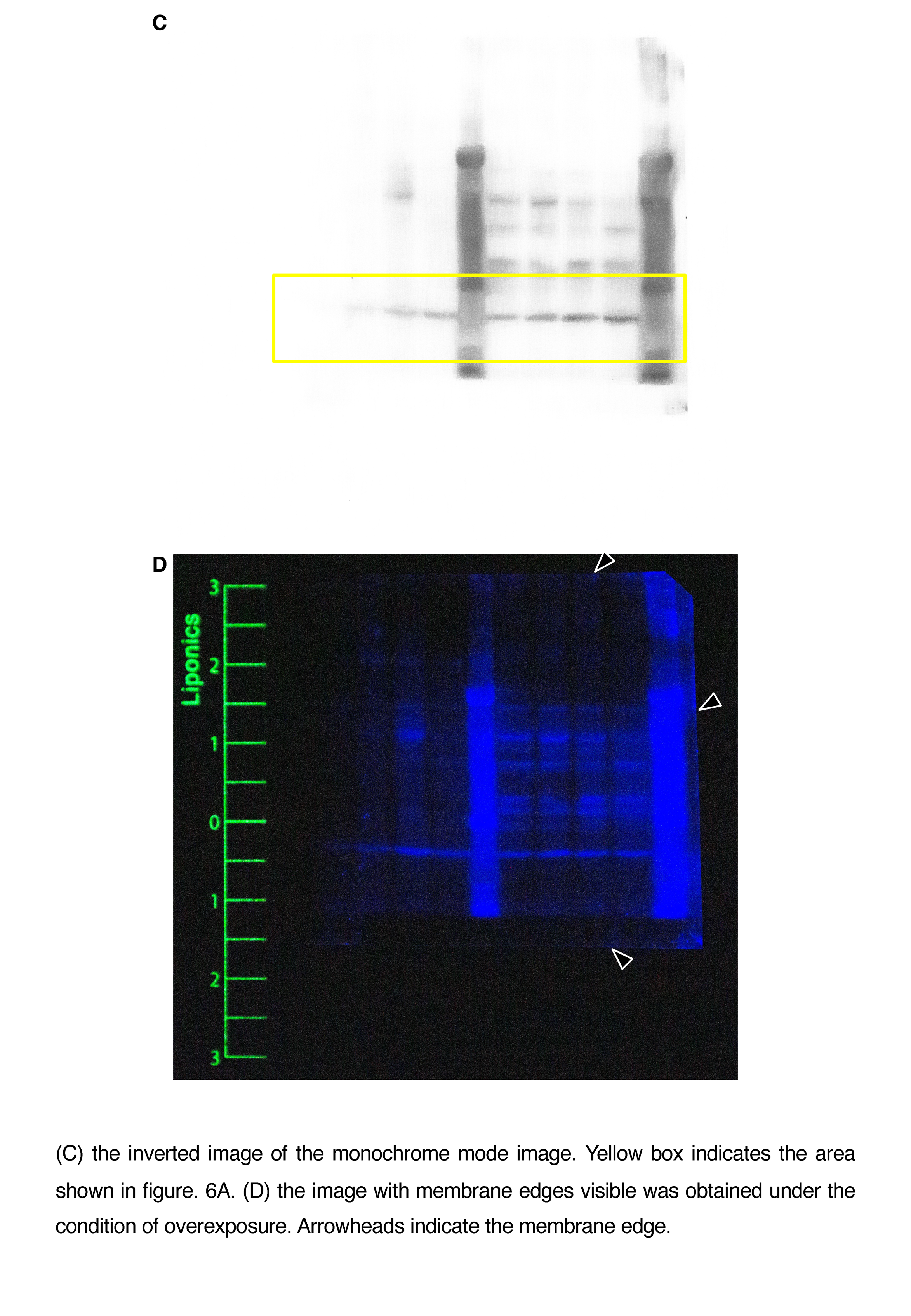

Supplement: Supplementary file 3 — Supplementary Information 3. [file 41598_2023_45902_MOESM3_ESM.tif]

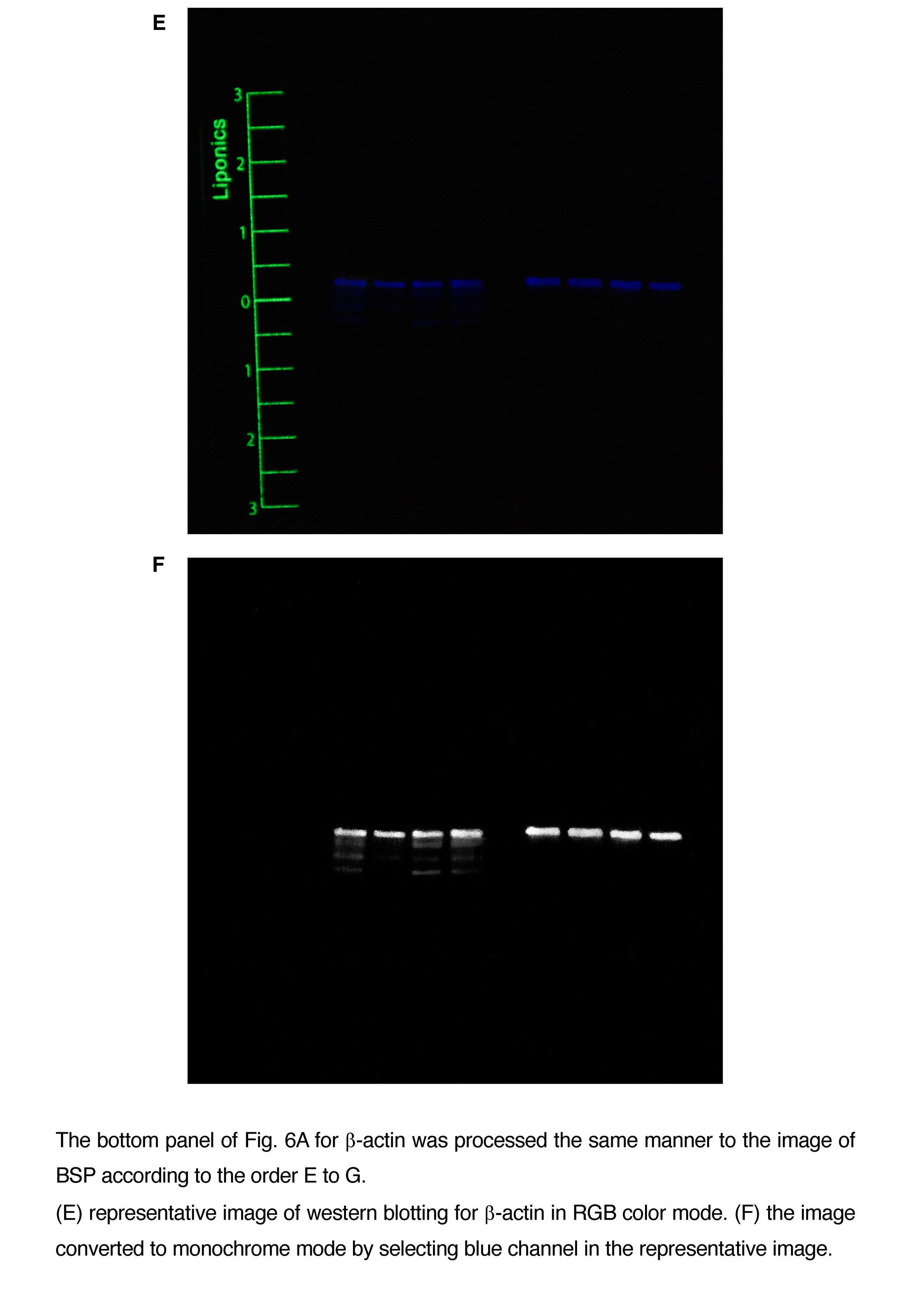

Supplement: Supplementary file 4 — Supplementary Information 4. [file 41598_2023_45902_MOESM4_ESM.tif]

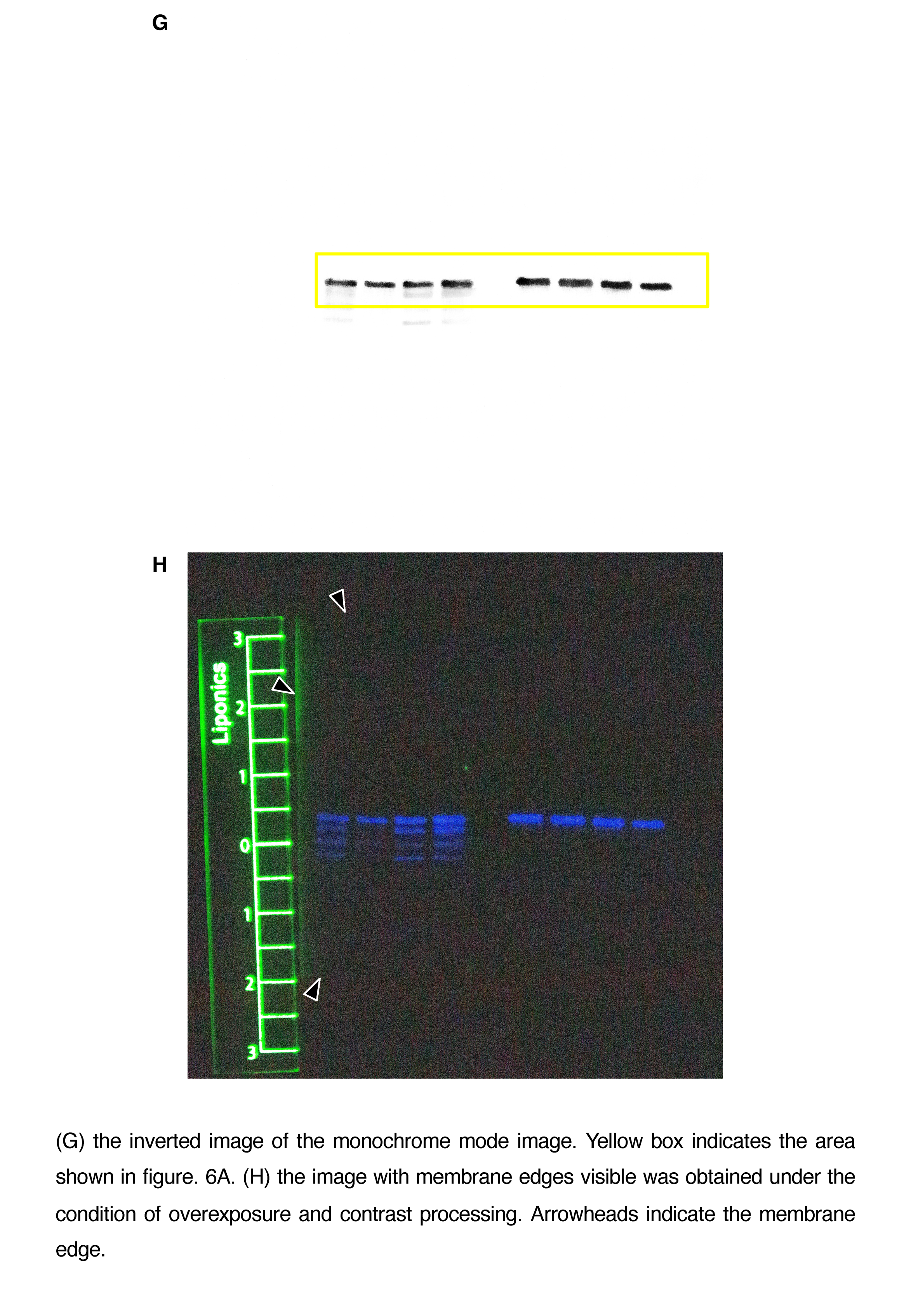

Supplement: Supplementary file 5 — Supplementary Information 5. [file 41598_2023_45902_MOESM5_ESM.tif]

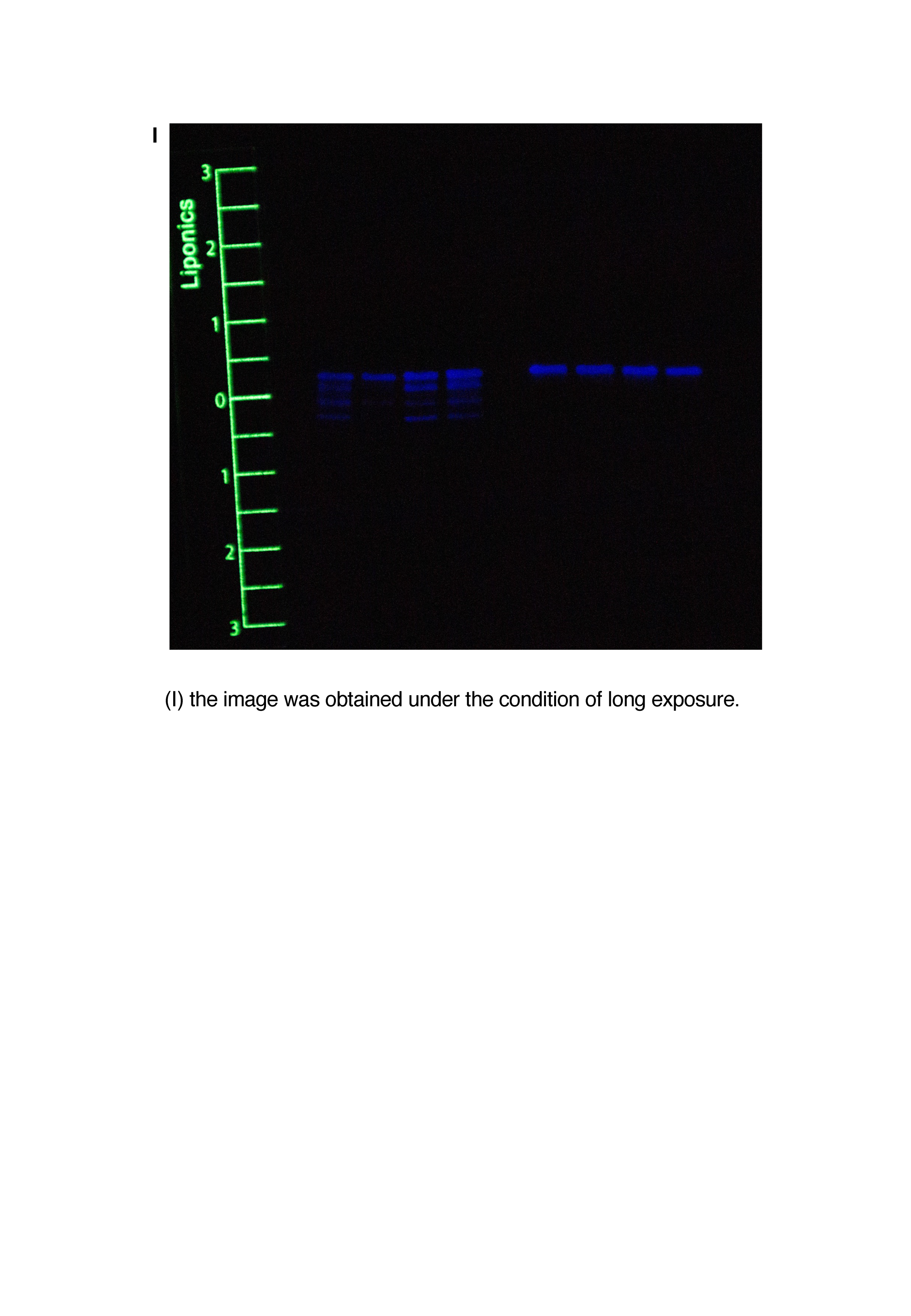

Supplement: Supplementary file 6 — Supplementary Information 6. [file 41598_2023_45902_MOESM6_ESM.tif]

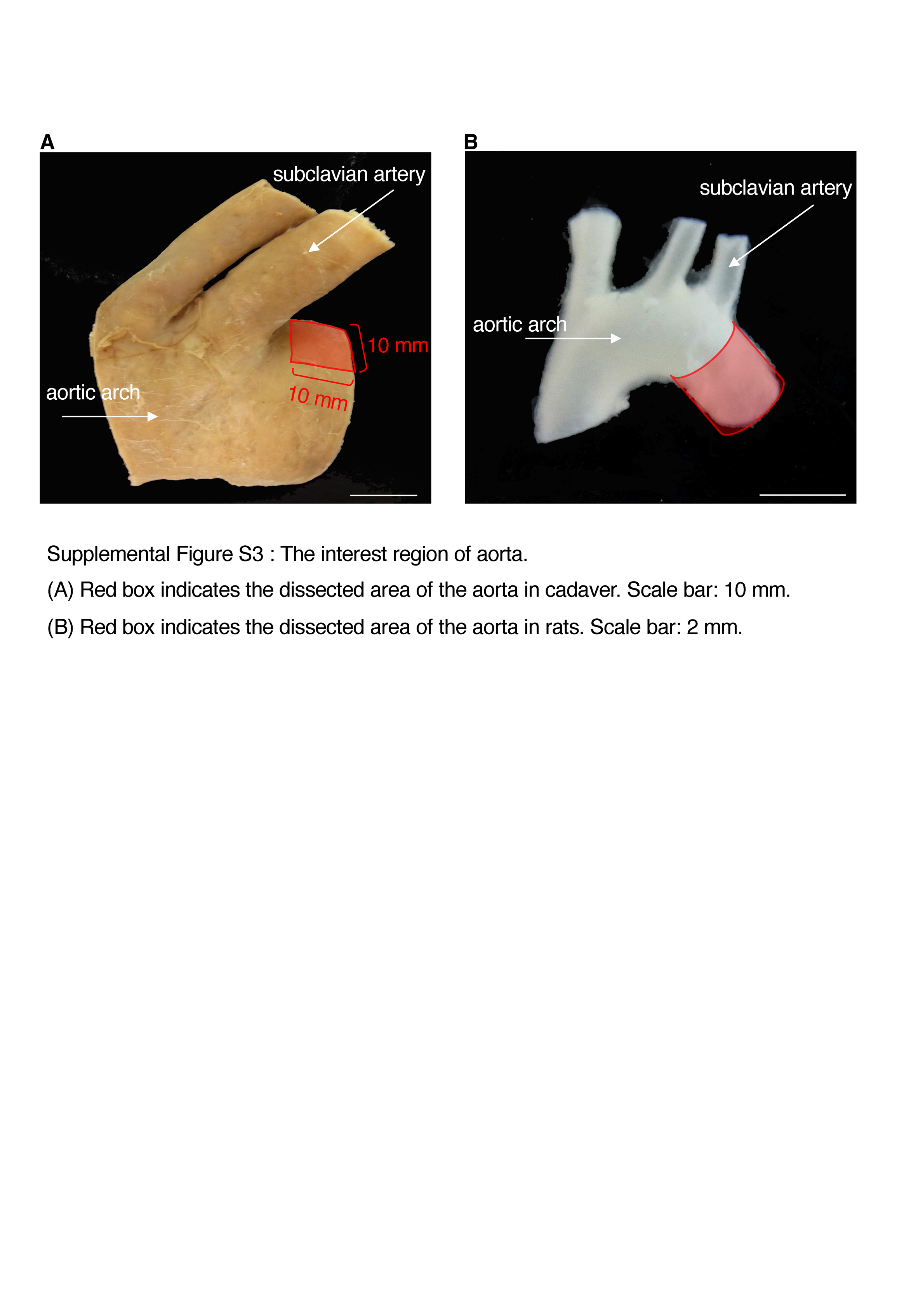

Supplement: Supplementary file 7 — Supplementary Information 7. [file 41598_2023_45902_MOESM7_ESM.tif]

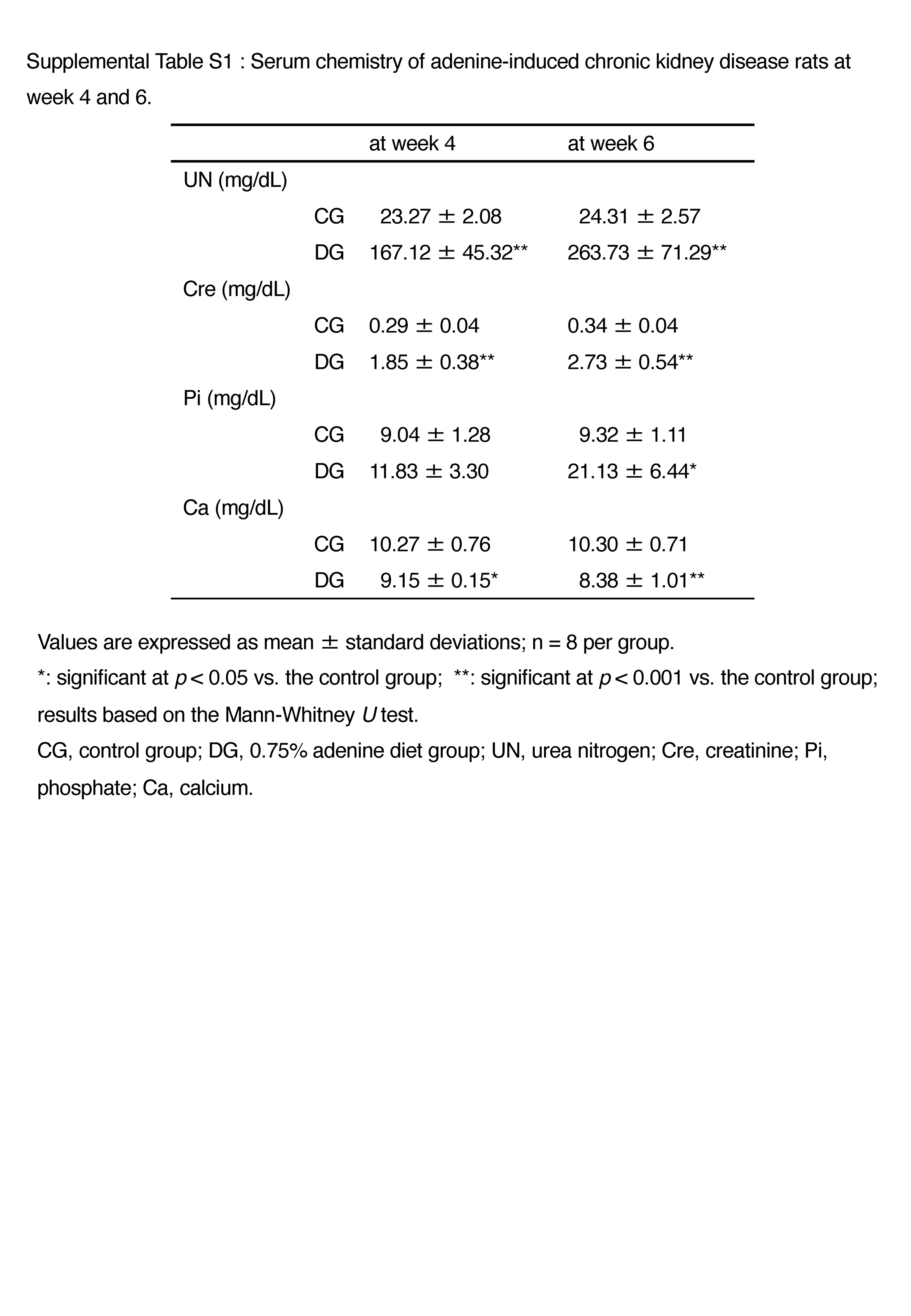

Supplement: Supplementary file 8 — Supplementary Information 8. [file 41598_2023_45902_MOESM8_ESM.tif]

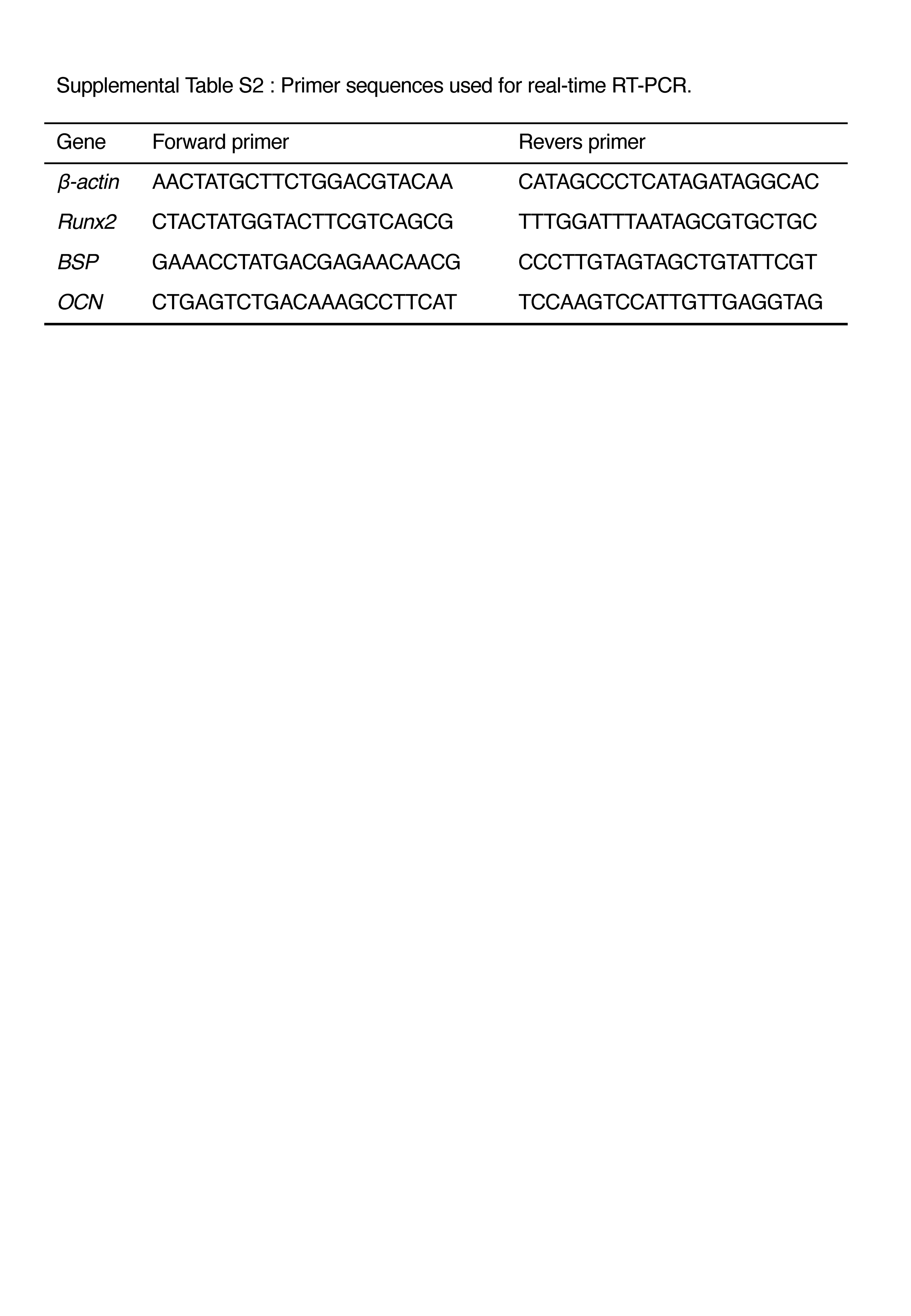

Supplement: Supplementary file 9 — Supplementary Information 9. [file 41598_2023_45902_MOESM9_ESM.tif]
